# Supplementary material for: Characterization of SlBAG Genes from Solanum lycopersicum and Its Function in Response to Dark-Induced Leaf Senescence
Source: Plants (Basel). 2021 May 10;10(5):947. doi: 10.3390/plants10050947 (PMC8151600; doi:10.3390/plants10050947)
Supplement: Supplementary file 1 [file plants-10-00947-s001.zip › Supplementary- data.pdf]

Supplementary data

Table S1 Primers used in this study

Gene name	Forward primer (5' to 3')	Reverse primer (5' to 3')	Primers used for
SAG	CTGTTACCCCTATCAAGGACCAAAT	TCGCCTTCGACATCACAATC	qPCR analysis
NAP2	GAGGTTGGATGATTGGGTACTTT	TTTGAGGTTCTTGTGTGTCTTCTTC	
SGR1	TATCCAGAGTTACAAGAAGC	CCACCAGAAGAAGATGAG	
PPH	GGAGGGAGCAAGTACGCTAT	AAGGCGTAGCATTCAGCAAG	
PAO	AAATGCCCTCATCGTCTTGC	AGCACAAGCTCTTGGAGACT	
NYC1	CAGGCAGCTTCAATCATCCC	TAGGTCGGTTAGGACCATGC	
NOL	GAGGACGATGGACTTAAC	TGTTGTTGTTGTTGTTGTT	
SAG20	TTGCTCAATATGCTCTGT	TGCTTCGTTGTCTTCTAA	
BAG2	TAGAGATGAAAGGGAAAGG	GAATCAACTCCACGAATG	
BAG5b	GAGAGGTGGATGAGATTGAACG	GCTTCCGTTCAATCTCAT	
HSP70	ATCCCGGTATTCTTTCTGTCA	GAGCCTTATCTCCCAACTCT	
HSP90	GTCCAGCAAGAAGACGATG	GCAGCAAAGGTATTAGGGT	
actin	TGGTCGGAATGGGACAGAAG	CTCAGTCAGGAGAACAGGGT	
BAG2 OE	ttacaattaccatggggcgcgccATGGAGTATCCATTATACGGAAGCT	aacatcgtatgggtaggtaccTGACATTTTTGCTACAGAAAGAATCA	Overexpression construct
BAG5b OE	ttacaattaccatggggcgcgccATGGAGAATCTCTTCAATTGGTCC	aacatcgtatgggtaggtaccGCTGCCGGAAACAATGGAG	
pFGC5941	GATGTGACATCTCCACTGACGT	GGCGGTAAGGATCTGAGCTA	Sequencing and determination
BAG2-GFP	TacaaatctatctctctcgagATGGAGTATCCATTATACGGAAGCT	GgatccccgggtaccgagctcAGCAGTGTGCCTCTTCTTCTTCTT	Subcellular localization
BAG5b-GFP	tacaaatctatctctctcgagATGGAGAATCTCTTCAATTGGTCC	ggatccccgggtaccgagctcGCTGCCGGAAACAATGGAG	construct

Table S2 Information on the tomato *BAG* family

Protein[a]	Gene accession no.			Predicted protein properties					Chromosome no.	Previous name [f]
	Locus name [b]	Gene ID [c]	NCBI accession no.	MW [d]	pI [d]	aa	CDS	Subcellular localization [e]		
SlBAG1	Solyc03g026220	LOC101264896	XP_004234356.1	38253.42	9.52	341	1026	Nucleus	3	BAG1
SlBAG2	Solyc06g072430	LOC104648101	XP_010322968.1	33401.75	5.66	373	1122	Nucleus	6	unknown
SlBAG3a	Solyc06g035720	LOC101267811	XP_010321994.1	37561.69	9.45	333	1002	Nucleus	6	BAG3
SlBAG3c	Solyc08g080320	LOC101246459	XP_004245651.1	31386.25	9.70	284	855	Nucleus	8	BAG3
SlBAG4a	Solyc10g085290	LOC101258018	XP_004249607.1	41906.74	5.09	394	1185	Nucleus	10	BAG4
SlBAG4b	Solyc06g007240	LOC101243790	XP_004240412.1	27551.40	6.28	278	837	Nucleus	6	BAG4
SlBAG5a	Solyc04g014740	LOC109119998	XP_019068857.1	27264.02	5.83	216	651	Nucleus	4	BAG5a
SlBAG5b	Solyc10g084170	LOC101250069	XP_004249799.1	19428.32	10.26	170	513	Chloroplast	10	BAG5b
SlBAG6	Solyc01g095320	LOC101246665	XP_004229963.1	52352.28	5.30	1235	3708	Nucleus	1	BAG6
SlBAG7	Solyc03g083970	LOC101246514	XP_004234927.1	45279.10	9.43	395	1188	Nucleus	3	BAG7

[a] Protein names adopted in this study.

[b] Systematic gene designation given by the Sol Genomics Network (SGN).

[c] Gene ID designation from the NCBI database.

[d] Predicted protein molecular masses and isoelectric points acquired from ExPASy Bioinformatics Resource Portal (available at https://web.expasy.org/compute_pi/).

[e] Subcellular localization predicted using the Balanced Subcellular Localization Predictor (http://gpcr.biocomp.unibo.it/bacello/pred.htm).

[f] Previous names taken from the NCBI database.
